# International corporate tax avoidance and domestic government health expenditure

**DOI:** 10.2471/BLT.18.220897

**Published:** 2019-09-03

**Authors:** Bernadette Ann-Marie O’Hare

**Affiliations:** a School of Medicine, University of St Andrews, North Haugh, St Andrews, KY16 9TF, Scotland.

## Abstract

**Objective:**

To compare estimated losses from international corporate tax avoidance in individual countries and domestic government health expenditure, with reference to the annual threshold of 86 United States dollars (US$) per capita required to achieve universal health coverage.

**Methods:**

I obtained and compared estimates of international corporate tax avoidance and domestic government health expenditure for 2013.

**Findings:**

Data were available for 100 countries: 24 low-, 28 lower-middle-, 21 upper-middle- and 27 high-income countries. Domestic government health expenditure was under US$ 86 per capita in all 24 low-income countries and in 24 of 28 lower-middle-income countries. International corporate tax lost per capita was higher than domestic government health expenditure in 19 low-income and 10 lower-middle-income countries. If the revenue lost to tax avoidance were recouped and allocated to the health sector, average annual government health expenditure could increase from US$ 8 to US$ 24 per capita in the low-income countries studied and from US$ 54 to US$ 91 per capita in the lower-middle-income countries.

**Conclusion:**

Recouping losses due to international corporate tax avoidance and allocating them to the health sector would help low- and lower-middle-income countries achieve universal health coverage, a target of sustainable development goal (SDG) 3. Tackling tax avoidance requires cooperation between the governments of all countries, multinational corporations and investors, including private individuals. International cooperation to improve domestic resource mobilization is the focus of SDG target 17.1.

## Résumé

**Objectif:**

Comparer les pertes estimées résultant de l'évitement de l'impôt sur les sociétés au niveau international dans certains pays et leurs dépenses de santé au niveau national, en se référant au seuil annuel de 86 dollars des États-Unis (US$) par habitant nécessaires pour parvenir à une couverture sanitaire universelle.

**Méthodes:**

J'ai obtenu et comparé des estimations de l'évitement de l'impôt sur les sociétés au niveau international et des dépenses de santé au niveau national pour l'année 2013.

**Résultats:**

Des données étaient disponibles pour 100 pays: 24 pays à faible revenu, 28 à revenu moyen inférieur, 21 à revenu moyen supérieur et 27 à revenu élevé. Les dépenses de santé du gouvernement national étaient inférieures à 86 US$ par habitant dans les 24 pays à faible revenu et dans 24 des 28 pays à revenu moyen inférieur. La perte résultant de l'évitement de l'impôt sur les sociétés au niveau international par habitant était supérieure aux dépenses de santé du gouvernement national dans 19 pays à faible revenu et 10 pays à revenu moyen inférieur. Si les revenus perdus en raison de l'évitement fiscal étaient récupérés et alloués au secteur de la santé, la moyenne des dépenses de santé annuelles du gouvernement pourrait passer de 8 US$ à 24 US$ par habitant dans les pays à faible revenu étudiés, et de 54 US$ à 91 US$ par habitant dans les pays à revenu moyen inférieur.

**Conclusion:**

La récupération des pertes dues à l'évitement de l'impôt sur les sociétés à l'échelle internationale et leur allocation au secteur de la santé aideraient les pays à faible revenu et à revenu moyen inférieur à parvenir à une couverture sanitaire universelle, ce qui correspond à une cible de l'objectif de développement durable (ODD) 3. La lutte contre l'évitement fiscal exige une coopération entre les gouvernements de tous les pays, les sociétés multinationales et les investisseurs, y compris les particuliers. La coopération internationale pour améliorer la mobilisation des ressources nationales est au cœur de la cible 17.1 des ODD.

## Resumen

**Objetivo:**

Comparar las pérdidas estimadas por la evasión del impuesto de sociedades internacional en los distintos países y el gasto sanitario de los gobiernos nacionales, con referencia al umbral anual de 86 dólares estadounidenses (USD) per cápita necesario para lograr la cobertura sanitaria universal.

**Métodos:**

Se obtuvieron y compararon estimaciones de la evasión del impuesto de sociedades internacional y del gasto sanitario de los gobiernos nacionales para 2013.

**Resultados:**

Los datos estaban disponibles para 100 países: 24 países de ingresos bajos, 28 de ingresos medios-bajos, 21 de ingresos medios-altos y 27 de ingresos altos. El gasto sanitario de los gobiernos nacionales fue inferior a 86 USD per cápita en los 24 países de bajos ingresos y en 24 de los 28 países de ingresos medios-bajos. La pérdida de impuestos de sociedades internacionales per cápita fue mayor que el gasto sanitario de los gobiernos nacionales en 19 países de ingresos bajos y 10 de ingresos medios-bajos. Si los ingresos perdidos por la evasión de impuestos se recuperaran y se asignaran al sector de la salud, el gasto público anual medio en salud podría aumentar de 8 a 24 USD per cápita en los países de bajos ingresos estudiados, y de 54 a 91 USD per cápita en los países de ingresos medios-bajos.

**Conclusión:**

La recuperación de las pérdidas debidas a la evasión del impuesto de sociedades internacional y su asignación al sector de la salud ayudaría a los países de ingresos bajos y medios-bajos a lograr la cobertura sanitaria universal, una meta del objetivo 3 del desarrollo sostenible (sustainable development goal, SDG). La lucha contra la evasión de impuestos requiere la cooperación entre los gobiernos de todos los países, las empresas multinacionales y los inversores, incluidos los particulares. La cooperación internacional para mejorar la movilización de recursos nacionales es el objetivo del SDG 17.1.

## ملخص

**الغرض:**

مقارنة الخسائر التقديرية نتيجة تجنب ضريبة المؤسسات الدولية في كل بلد على حدة، والإنفاق الحكومي المحلي على الصحة، مع الإشارة إلى الحد السنوي البالغ 86 دولاراً أمريكياً للفرد، والمطلوب لتحقيق التغطية الصحية الشاملة.

**الطريقة:**

لقد حصلت على التقديرات لتجنب ضريبة المؤسسات الدولية، والإنفاق الحكومي المحلي على الصحة لعام 2013، وقمت بالمقارنة بينها.

**النتائج:**

كانت البيانات متوفرة لعدد 100 دولة:24 دولة ذات دخل منخفض، و28 دولة ذات دخل متوسط، و21 دولة ذات دخل متوسط أعلى، و27 دولة ذات دخل مرتفع.كان الإنفاق الحكومي المحلي على الصحة أقل من 86 دولاراً أمريكياً للفرد في كل الدول الـ 24 ذات الدخل المنخفض، وفي 24 دولة من إجمالي الـ 28 دولة ذات الدخل المتوسط الأقل.كانت ضريبة المؤسسات الدولية الضائعة بالنسبة للفرد أعلى من الإنفاق الحكومي المحلي على الصحة في 19 دولة ذات دخل منخفض، و10 دول ذات دخل متوسط أقل.إذا كان العائد الضائع بسبب تجنب الضريبة، قد تم استرداده وتخصيصه للقطاع الصحي، يمكن أن يزيد متوسط ​​الإنفاق الحكومي السنوي على الصحة من 8 دولارات إلى 24 دولاراً للفرد في الدول ذات الدخل المنخفض الخاضعة للدراسة، ومن 54 دولاراً أمريكياً إلى 91 دولاراً أمريكياً للفرد في الدول ذات الدخل المتوسط الأقل.

**الاستنتاج:**

إن استرداد الخسائر الناتجة عن تجنب ضريبة المؤسسات الدولية، وتخصيصها للقطاع الصحي سوف يساعد الدول ذات الدخل المنخفض، والدول ذات الدخل المتوسط الأقل، في تحقيق ​​تغطية صحية شاملة، وهو الغاية المنشودة من هدف التنمية المستدامة الثالث (SDG). يتطلب تعقب تجنب الضريبة تعاونًا بين الحكومات في كل الدول، والمؤسسات متعددة الجنسيات، والمستثمرين، بما يشمل الأفراد ذاتهم.ويمثل التعاون الدولي لتحسين تعبئة الموارد المحلي محورًالهدف التنمية المستدامة (SDG) رقم 17.1.

## 摘要

**目的:**

参照实现全民健康覆盖所需的人均 86 美元 (US$) 的年度最低要求，比较各国国际企业避税行为造成的预计损失和国内政府卫生支出。

**方法:**

我们搜集并比较了 2013 年国际企业避税和国内政府卫生支出的预估值。

**结果:**

提供了 100 个国家/地区的数据：24 个低收入国家、28 个中低收入国家、21 个中高收入国家和 27 个高收入国家。所有 24 个低收入国家以及 28 个中低收入国家中的 24 个国家的国内政府卫生支出低于 86 美元的人均标准。在 19 个低收入国家和 10 个中低收入国家中，国际企业人均税收损失高于国内政府卫生支出。如果将国际企业因避税造成的税收损失收回并分拨给卫生部门，则本文所研究的低收入国家的年均卫生支出可以从人均 8 美元增加到 24 美元，中低收入国家的年均卫生支出可以从人均 54 美元增加到 91 美元。

**结论:**

将国际企业因避税造成的税收损失收回并分拨给卫生部门有助于低收入和中低收入国家实现全民健康覆盖，即可持续发展目标 (SDG) 3 的目标。治理避税问题需要各国政府、跨国公司以及包括个人在内的投资者之间的通力合作。加强国内资源调动的国际合作是可持续发展目标 17 的重点。

## Резюме

**Цель:**

Сравнение оценки ущерба от международного уклонения от уплаты налога на прибыль предприятий и организаций в отдельных странах и объемов внутренних государственных расходов на здравоохранение в сравнении с ежегодным порогом в 86 долларов США на душу населения, необходимым для достижения всеобщего охвата услугами здравоохранения.

**Методы:**

Автор получил и сравнил оценочные данные о суммах международного уклонения от уплаты налога на прибыль предприятий и организаций и внутренних государственных расходах на здравоохранение по состоянию на 2013 год.

**Результаты:**

Были доступны данные по 100 странам: 24 страны с низким уровнем доходов, 28 стран с уровнем доходов от низкого к среднему, 21 страна с уровнем доходов от среднего к высокому и 27 стран с высоким уровнем доходов. Внутренние государственные расходы на здравоохранение были ниже 86 долларов США на душу населения во всех 24 странах с низким уровнем доходов и в 24 из 28 стран с уровнем доходов от низкого к среднему. Объемы ущерба от международного уклонения от уплаты налога на прибыль предприятий и организаций были выше, чем внутренние государственные расходы на здравоохранение, в 19 странах с низким уровнем доходов и в 10 странах с уровнем доходов от низкого к среднему. Если бы прибыль, потерянную в результате уклонения от уплаты налогов, удалось бы вернуть в страну и передать в сектор здравоохранения, можно было бы повысить среднегодовые расходы на душу населения с 8 до 24 долларов США в исследованных странах с низким уровнем доходов и с 54 до 91 доллара США в странах с уровнем доходов от низкого к среднему.

**Вывод:**

Возврат в страну прибыли, потерянной в результате международного уклонения от уплаты налога на доход предприятий и организаций, и ее ассигнование на нужды сектора здравоохранения могли бы помочь странам с низким и средне-низким уровнем доходов добиться всеобщего охвата услугами здравоохранения, то есть достичь цели номер 3 в области устойчивого развития (SDG). Борьба с уклонением от уплаты налогов требует совместных усилий правительств всех стран, многонациональных корпораций и инвесторов, в том числе частных лиц. Международное сотрудничество для улучшения мобилизации внутренних ресурсов стран является главной частью цели SDG 17.1.

## Introduction

Half the world’s population does not have essential health services and many more do not have access to clean water, sanitation or education, all of which can contribute as much to health outcomes as health care.^1^^–^^3^ Many of the sustainable development goals (SDGs) address these issues and include targets for health coverage and determinants of health.^4^ In particular, SDG 3 focuses on good health and well-being, with target 3.8 being the achievement of universal health coverage (UHC) for all, without financial risk, by 2030. However, universal coverage does not imply that all health interventions should be free. Instead, the intention is to reduce the fraction of the population that spends a substantial portion of their household income on health and is, thus, at risk of financial hardship. Outcome indicators associated with target 3.8 reflect health coverage and the proportion of households with catastrophic health spending,^1^^,^^5^ which occurs when out-of-pocket payments on health exceed a certain percentage of household income, either 10% or 25%(Box 1).^7^ When out-of-pocket payments make up less than 20% of a country’s total health expenditure, catastrophic spending for households is much less likely. However, in low-income countries, these payments account for 42% of total health expenditure; in lower-middle-income countries, the proportion is 56%.^4^

Box 1 **Glossary of financial terms**

**Government health expenditure:** financial outlay by government entities to purchase health-care services and goods, which can come from both domestic and external funding sources (mainly grants administered by government or loans channelled through the national budget) – often reported as a percentage of GDP.

**Domestic government health expenditure:** government health expenditure that comes from domestic sources only.

**Household out-of-pocket payments:** a household’s outlay on health care, including direct payments to public and private health-care providers and non-profit institutions and non-reimbursable cost-sharing, such as deductibles, co-payments and fees for services.

**Total health expenditure:** national expenditure on health care, including government health expenditure, out-of-pocket payments, prepaid private health insurance (by families and employers) and development assistance for health.

**Tax avoidance:** the legal practice of seeking to minimize a tax bill by taking advantage of a loophole or adopting an unintended interpretation of the tax code.

**Base erosion and profit-shifting:** tax-avoidance strategies that exploit gaps and mismatches in tax rules to artificially erode the tax base in a country and shift profits to low- or no-tax locations.

**Tax evasion:** intentionally defrauding revenue authorities rather than using loopholes or legal methods.

**Illicit financial flows:** (i) mostly commercial tax avoidance or evasion (sometimes called ‘legal capital’ illicit financial flows, for example from tax abuse involving income or profits that were originally earned legitimately); and (ii) about one quarter to one third resulting from laundering the proceeds of criminal activity and a small percentage due to corruption or theft (sometimes called ‘illegal capital’ illicit financial flows).

**Tax haven or low-tax jurisdiction:** a country or place with a low tax rate, in which people often choose to live or register companies to avoid paying higher tax in their own countries. Tax havens undermine the finances of countries where real economic activity takes place by providing some combination of secrecy and manipulative tax rules that make it possible to book and hide business that should be taxed or regulated elsewhere. (An estimated 10% of the world’s GDP is held offshore).^6^

GDP: gross domestic product.

Even though all countries have pooling mechanisms for health, such as health ministry budgets and prepaid health insurance, out-of-pocket payments continue to be critical. The hope is that establishing UHC will reduce poverty (SDG 1), given that 12% of the world’s population spend more than 10% of their household income on health.^1^ Moreover, a reduction in the household budget allocated to health could boost educational attainment (SDG 4), which in turn could increase gender equity (SDG 5), stimulate economic growth (SDG 8) and promote a just and inclusive society (SDG 16).^1^

The achievement of UHC will largely be determined by the capacity of governments to provide, or regulate the private provision of, health services. In 2009, the World Health Organization (WHO) estimated that providing UHC would require the expenditure of at least 54 United States dollars (US$) per capita each year.^8^ In 2012, the figure was updated to at least US$ 86 per capita, with the added condition that a country should spend at least 5% of its gross domestic product (GDP) on health.^8^ More recent recommendations are for a minimum of US$ 112 per capita (at 2014 values) and 7.5% of GDP allocated to health by 2030. This increase was due to higher current baseline spending, more ambitious targets and inclusion of the cost of tackling noncommunicable diseases.^9^

Many people believe that, if UHC is to be provided sustainably, it should be funded domestically. This approach may be feasible because domestic financing is responsible for 70% of total health expenditure in low-income countries and 86% in lower-middle-income countries.^8^ Most funding comes from out-of-pocket payments because many governments spend small amounts on health,^10^ often only a fraction of the US$ 86 per capita required annually and well below 5% of GDP.^11^ Moreover, domestic government health expenditure is a smaller percentage of overall government expenditure in lower-income countries than in wealthier countries. For example, in 2015 the average proportion of the overall government budget allocated to health was 5% in lower-middle-income countries, 10% in upper-middle-income countries and 23% in high-income countries.^12^

Government health expenditure comes from public or pooled funds, from either general government revenue or social health insurance contributions.^13^ However, collecting insurance contributions from unwaged citizens working in the informal sector can be difficult and general government revenue is a more likely source of funding in many countries. As government revenue increases, so does health expenditure. In low-income countries, for instance, a 10% increase in tax revenue has been estimated to lead to a 17% increase in government health expenditure.^14^ However, in 2018, the average general government revenue per capita was only around US$ 100 in low-income countries and around US$ 400 in lower-middle-income countries.^15^ Therefore, spending more on health, to provide UHC, needs higher government revenue.

In low- and lower-middle-income countries, 70% of general government revenue comes from taxes;^16^ the balance is from grants and non-tax revenues, such as royalties from natural resource extraction. As a percentage of GDP, tax is much higher in high-income countries, at around 40%, than in low- and lower-middle-income countries, where it is around 20%, although it is increasing.^17^ This large gap in tax receipts may, for example, be due to: (i) international corporate tax avoidance; (ii) failure to tax the informal sector or high-net-worth individuals; or (iii) tax incentives granted to the corporate sector by governments to attract foreign investment.^16^ Other sources of lost government revenue, which are beyond the scope of this study, include theft from the public purse and debt repayment.^18^

Tax avoidance in the informal sector and granting tax incentives are within the remit of governments and could be curtailed.^19^ In many low- and lower-middle-income countries, the informal sector accounts for 40% of GDP. Failure to tax this sector is partly logistical and partly a lack of political will. As in all countries, the wealthy and the political elite may have an undue influence on tax policies and their administration.^16^^,^^20^ Tax incentives or waivers are often given to attract foreign investment, but there is no evidence that these drive economic growth (Stausholm, Copenhagen Business School, unpublished observations, 2017) and signing tax treaties with tax havens or low-tax jurisdictions has a substantial impact on revenue.^21^

Corporate tax is an indispensable source of income for all countries, particularly low- and lower-middle-income countries, where it accounts for 20% of government revenue. In wealthy countries, the proportion is 10%.^20^ However, corporate tax avoidance has been estimated at US$ 500 to 650 billion internationally each year, with one third occurring in low- and lower-middle-income countries where such tax avoidance has a disproportionately large impact on the government revenue needed for public services.^22^^,^^23^ In these countries, the income generated by corporations within their borders is one of the few additional sources of public funding realistically accessible over the short to medium term. Although recouping international corporate tax may not automatically result in improved public services, it will increase the chance of improvement.^24^

The responsibility for international corporate tax avoidance lies with a broad group of actors, including: (i) multinational corporations themselves; (ii) governments of high-income countries, where parent companies are usually based and therefore regulated; and (iii) governments of low- and lower-middle-income countries.^25^ In theory, corporate income tax is payable when a multinational corporation begins to realize profits. However, there are techniques for reducing a corporation’s tax base (referred to as base erosion and profit-shifting); for example, overpricing costs and under-reporting profits (i.e. mispricing). Mispricing can also include overpayment for intangible services, such as intellectual property or managerial support. Furthermore, when borrowing from a related entity (i.e. a business with a separate legal existence) in a low-tax jurisdiction, repayment with interest allows profits to be shifted to that jurisdiction. The rules governing the selling of goods or services between related entities include the principle that multinational corporations theoretically charge related entities the same price as they would an independent company.However, this principle is frequently circumvented, often by using tax havens.^26^ The existence of differential tax rates between countries provides an incentive for multinational corporations to move profits out of high-tax jurisdictions into tax havens by setting up a related entity or subsidiary. In addition, tax treaties, which rarely benefit low-income countries, are commonly used to minimize tax by diverting profits through an entity based in a country that has a favourable treaty in place.^27^

Given the potential of UHC to reduce poverty and increase economic growth, it is vital to consider how health care can be sustainably financed. As most general government revenue comes from taxes, identifying the reasons for gaps in tax revenue is a priority. National governments have the power to reduce some gaps, for example, by curtailing tax incentives. Other gaps are the responsibility of the international community, which has committed to achieving UHC within SDG 3. In addition, SDG 17.1 target calls for international efforts to strengthen domestic resource mobilization.

The aims of this study were to compare losses from international corporate tax avoidance in individual countries and domestic government health expenditure, with reference to the recommended annual threshold of US$ 86 per capita required for UHC, and to discuss how such tax avoidance could be curtailed.

## Methods

A glossary of financial terms used here are presented in Box 1. I performed a cross-sectional study of countries at all income levels to compare losses from government revenue due to international corporate tax avoidance with domestic government health expenditure. As tax avoidance tends to be hidden, indirect methods have evolved to estimate its magnitude. The World Bank residual model, for example, examines the difference between funds entering a country and that country’s total expenditure. Any shortfall in expenditure is regarded as reflecting illicit financial flows. Other approaches examine trade data using, for example, a trade-mirror approach: mismatches are sought between what a country reports it exports and what the corresponding importing countries report.^28^^,^^29^ This method has been criticized for making too many assumptions.^30^ Rather than looking at financial flows, some researchers have studied the wealth held in tax havens. However, limited data are available.

The data used here were taken from an analysis done by Cobham and Janský, who repeated an earlier analysis performed by the International Monetary Fund.^22^^,^^23^ Cobham and Janský estimated the tax revenue an individual country would gain in 2013 if the opportunity for profit-shifting to tax havens were eliminated by raising the average corporate income tax rate in tax havens to the domestic rate. The extent to which differentials in tax rates drive differences in tax revenues was estimated using regression analysis, with the domestic corporate income tax rate as the dependent variable and the corresponding tax rate in tax havens as an independent variable. There were several differences between Cobham and Janský’s analysis and the International Monetary Fund’s study: (i) government revenue data were obtained from the United Nations University’s World Institute for Development Economics Research (UNU-WIDER) and cover more years for some countries;^15^ (ii) Bermuda, Ireland, Luxembourg, the Netherlands, Singapore and Switzerland were included as tax havens; and (iii) critically, results were presented for individual countries. Nevertheless, the findings of both studies were similar. Tax avoidance data were available as a percentage of GDP and were converted into US$ per capita at the dollar’s 2010 value.

I obtained estimates of national health expenditure, including total health expenditure and domestic government health expenditure, from WHO’s expenditure database for 2013 to allow direct comparison with tax avoidance data.^31^ A full description of the data and data sources is available from the author on request.

## Results

**Fig. 1:**
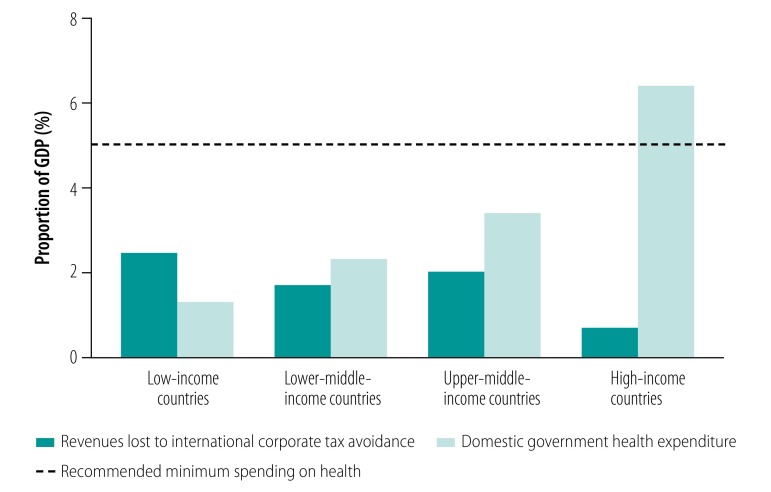
Revenues lost to international corporate tax avoidance in comparison to domestic government health expenditure, by country income level, 2013

Data on international corporate tax avoidance in 2013 were available for 100 countries: 24 low-, 28 lower-middle-, 21 upper-middle- and 27 high-income countries. Fig. 1 shows that domestic government health expenditure was well below 5% of GDP in low-, lower-middle- and upper-middle-income countries. In low-income countries, average corporate tax avoidance was larger than domestic government health expenditure as a percentage of GDP. In addition, Table 1 shows that the average government revenue in low-income countries was just over US$ 100 per capita each year and that corporate tax avoidance per capita (average: US$ 15.60) was almost twice domestic government health expenditure (average: US$ 8.35). 

GDP: gross domestic product.

**Table 1:** Estimated revenues lost to international corporate tax avoidance in comparison to domestic government health expenditure, by country income level,2013

Parameter	No. of observations	Value in US$^a^	
		Mean (SD)	Range
**Low-income countries (*n* = 24)**			
Revenues lost to corporate tax avoidance per capita	24	15.6 (13.1)	3.0–65.1
Domestic government health expenditure per capita	24	8.4 (4.6)	1.5–22.2
Government revenue per capita	22	107.7 (58.9)	16.9–261.2
**Lower-middle-income countries (*n* = 28)**			
Revenues lost to corporate tax avoidance per capita	28	37.4 (29.0)	6.4–98.2
Domestic government health expenditure per capita	28	54.0 (48.5)	3.5–186.0
Government revenue per capita	25	416.5 (276.4)	81.4–1186.0
**Upper-middle-income countries (*n* = 21)**			
Revenues lost to corporate tax avoidance per capita	21	134.2 (117.2)	1.1–472.9
Domestic government health expenditure per capita	21	234.2 (119.8)	79.0–560.1
Government revenue per capita	19	1 647.0 (624.5)	811.5–2 903.0
**High-income countries (*n* = 27)**			
Revenues lost to corporate tax avoidance per capita	27	194.7 (227.9)	7.0–1 031.0
Domestic government health expenditure per capita	27	2 531.0 (1 588.0)	276.7–5 650.0
Government revenue per capita	26	15 526.0 (10 056.0)	2 211.0–46 053.0

SD: standard deviation; US$: United States dollar.

^a^ All amounts are expressed in United States dollars at their 2010 value.

**Fig. 2:**
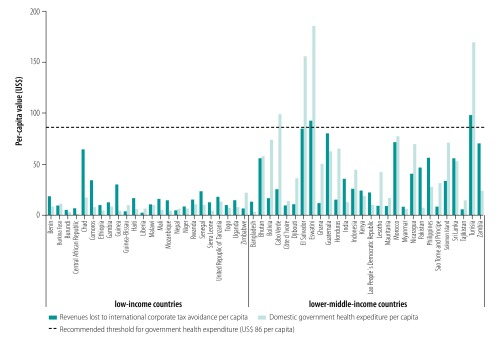
Per-capita revenues lost to international corporate tax avoidance in comparison to domestic government health expenditure, low- and lower-middle-income countries, 2013

If the lost international corporate tax were recouped and reallocated to the health sector, the average government health expenditure in the low-income countries studied could increase from around US$ 8 per capita annually to around US$ 24 per capita and that in lower-middle-income countries could increase from around US$ 54 to around US$ 91 per capita, above the recommended annual threshold of US$ 86 per capita. However, the data available indicate that domestic government health expenditure in 2013 was below US$ 86 in all 24 low-income countries and in 24 of the 28 lower-middle-income countries studied (Fig. 2). Moreover, international corporate tax avoidance per capita was greater than domestic government health expenditure per capita in 19 low-income countries and 10 lower-middle-income countries.

US$: United States dollar.

Note: All amounts are expressed in United States dollars at their 2010 value.

## Discussion

This analysis found that, on average in 2013, government revenue lost to international tax avoidance in low-income countries was greater than domestic government health expenditure when assessed either as a percentage of GDP or on a per capita basis. In 2013, per-capita domestic government health expenditure was far below the annual US$ 86 target for achieving UHC in all low-income countries studied and in most lower-middle-income countries.

The main limitation of the study was the availability and reliability of tax avoidance data. Currently, the magnitude of tax avoidance can be estimated only indirectly, but increased transparency, new methods and new data leaks should lead to improvements in the future.^6^ The secondary data used here were consistent with those in an earlier analysis, with the advantage that estimates are provided for individual countries. Although indirect methods will always be questioned, the findings of the two studies give some indication of the scale and potential of the health-care gains that could result from increased domestic resource mobilization. These results will add to current pressure for greater transparency and better tax avoidance data.

This study showed the potential gains of curtailing corporate tax avoidance in terms of the estimated cost of UHC and current government spending on health. One cannot assume, however, that all recovered revenues would be channelled towards the health sector because each country allocates revenue in accordance with its own priorities and the bargaining power of different ministries. One study of 25 countries that received debt relief found that expenditure on education, preventative health care and infrastructure increased on average by 2.5% of GDP as a result.^32^ In addition, recouping corporate tax revenues may decrease reliance on other sources of taxation, such as consumption taxes, which have been considered regressive and have been associated with increased child mortality. Less reliance on consumption taxes may indirectly improve health.^33^ Moreover, allocating recouped revenues to other sectors of society, for example the educational or agricultural sector, may have a greater impact on economic growth. A study showed that an increase in spending on education of 1.0% of GDP was associated with an increase in per-capita GDP of 1.4%; by comparison, a similar increase in health spending was associated with a 0.5% increase in per-capita GDP.^34^ Other considerations that could influence the allocation of recouped revenues are whether revenue generation and allocation in a country are decentralized and whether the revenue from international corporations is mainly generated in one part of the country.^35^

As countries grow economically, government health expenditure generally increases as a proportion of total health expenditure and out-of-pocket payments decrease. Reliance on out-of-pocket payments is slowly declining in all WHO regions as countries take actions to address catastrophic health spending: in low-income countries, out-of-pocket payments decreased from 54% of total health expenditure in 1995 to 42% in 2015.^36^ However, this decrease resulted from increased aid for health, rather than from greater domestic government health expenditure, which still accounted for only 25% of total health expenditure.^8^^,^^37^ Although I found that international corporate tax avoidance in low-income countries was almost twice domestic government health expenditure on average, even channelling all the revenue lost into the health sector would still result in government health expenditure being far below the US$ 86 threshold. Consequently, many countries will still rely on out-of-pocket payments for the foreseeable future and these payments will be essential for financing UHC. 

Governments could use the revenue recouped from corporate tax avoidance to increase the efficiency and quality of both public and private (including aid-funded) health facilities. In particular, substantial improvements could be achieved for relatively small amounts of money by regulating and managing performance and ensuring measures are in place to protect vulnerable patient groups from catastrophic health costs. Private clinics could be offered incentives to prioritize government-determined health goals, thereby maximizing the benefit of both public and private investment in health. Beneficiaries of these incentives could agree to regular evaluation by government agencies, which would increase the quality and efficiency of the private market and improve transparency. One strategy for improving the quality and efficiency of health systems is performance-based financing, which has been embraced by many countries. This strategy can promote dialogue between health-care purchasers, providers and other stakeholders.^38^ Despite some criticism,^39^ many experts working in Africa have found this strategy useful when adapted to local needs.^40^ A small amount of recouped public money could drive a virtuous circle, in which improved public services increase tax morale (i.e. perceptions of and attitudes towards taxes) and generate more taxes, which can be used to strengthen public services further.^41^

One strategy for tackling international corporate tax avoidance, which has been proposed by the Organisation for Economic Co-operation and Development and the G20 group of nations, is to require large multinational corporations to report their economic activity, profits and taxes paid in individual countries to the revenue authority where the parent company is based. In 2015, a package of measures on base erosion and profit-shifting was agreed by 100 countries and jurisdictions.^42^ However, these measures were quickly seen to have failed in their central goal of ensuring better alignment between the place where a corporation’s activity occurs and the place where its taxable profits are declared. A successor framework has just been launched by the G20. For the first time, by using a formula, taxable profits could be directly apportioned between countries according to where the corporation’s activity (e.g. employment and sales) takes place.^43^^,^^44^

In addition, high-income countries, where most parent companies of multinationals are based, could sanction corporations that aggressively avoid tax in low- or lower-middle-income countries by withdrawing public procurement contracts, export credit guarantees and other forms of state support.^45^ Overseas development aid and expertise could be used to provide training for multinational corporations on the importance of business integrity and tax responsibility. Furthermore, multinational corporations could report their tax contributions publicly in their economic, social and governance reports, as favourable reports may attract ethical investors. Moreover, individual investors and pension holders could also contribute to human development by ensuring that their investments or pension funds are in multinational corporations that pay tax transparently.^25^

In conclusion, increased domestic government health expenditure is required to achieve UHC. Expenditure is needed for both the direct provision of health care and for ensuring quality by regulating public and private providers. Recouping government revenues lost to international corporate tax avoidance would be an important step in the right direction and is the responsibility of both national governments and the international community, which has committed to improving tax collection in accordance with SDG target 17.1.
